# Fostering open collaboration in drug development for paediatric brain tumours

**DOI:** 10.1042/BST20190315

**Published:** 2019-09-24

**Authors:** Jong Fu Wong, Elizabeth J. Brown, Eleanor Williams, Alex N. Bullock

**Affiliations:** Structural Genomics Consortium, University of Oxford, Old Road Campus, Roosevelt Drive, Oxford OX3 7DQ, U.K.

**Keywords:** BMP, brain tumour, cancer, drug development, glioma, kinase

## Abstract

Brain tumours have become the leading cause of child mortality from cancer. Indeed, aggressive brainstem tumours, such as diffuse intrinsic pontine glioma (DIPG), are nearly uniformly fatal. These tumours display a unique set of driver mutations that distinguish them from adult gliomas and define new opportunity for the development of precision medicines. The specific association of *ACVR1* mutations with DIPG tumours suggests a direct link to neurodevelopment and highlights the encoded bone morphogenetic protein receptor kinase ALK2 as a promising drug target. Beneficial effects of ALK2 inhibition have now been observed in two different *in vivo* models of DIPG. Nonetheless, such tumours present a huge challenge for traditional economic models of drug development due to their small market size, high failure rate, tumour location and paediatric population. Moreover, a toolkit of different investigational drugs may be needed to fully address the heterogeneity of these tumours in clinical trials. One new business model is suggested by M4K Pharma, a recent virtual start up that aims to align diffuse academic and industry research into a collaborative open science drug discovery programme. Fostering scientific collaboration may offer hope in rare conditions of dire unmet clinical need and provide an alternative route to affordable medicines.

## Introduction

**Figure 1.**
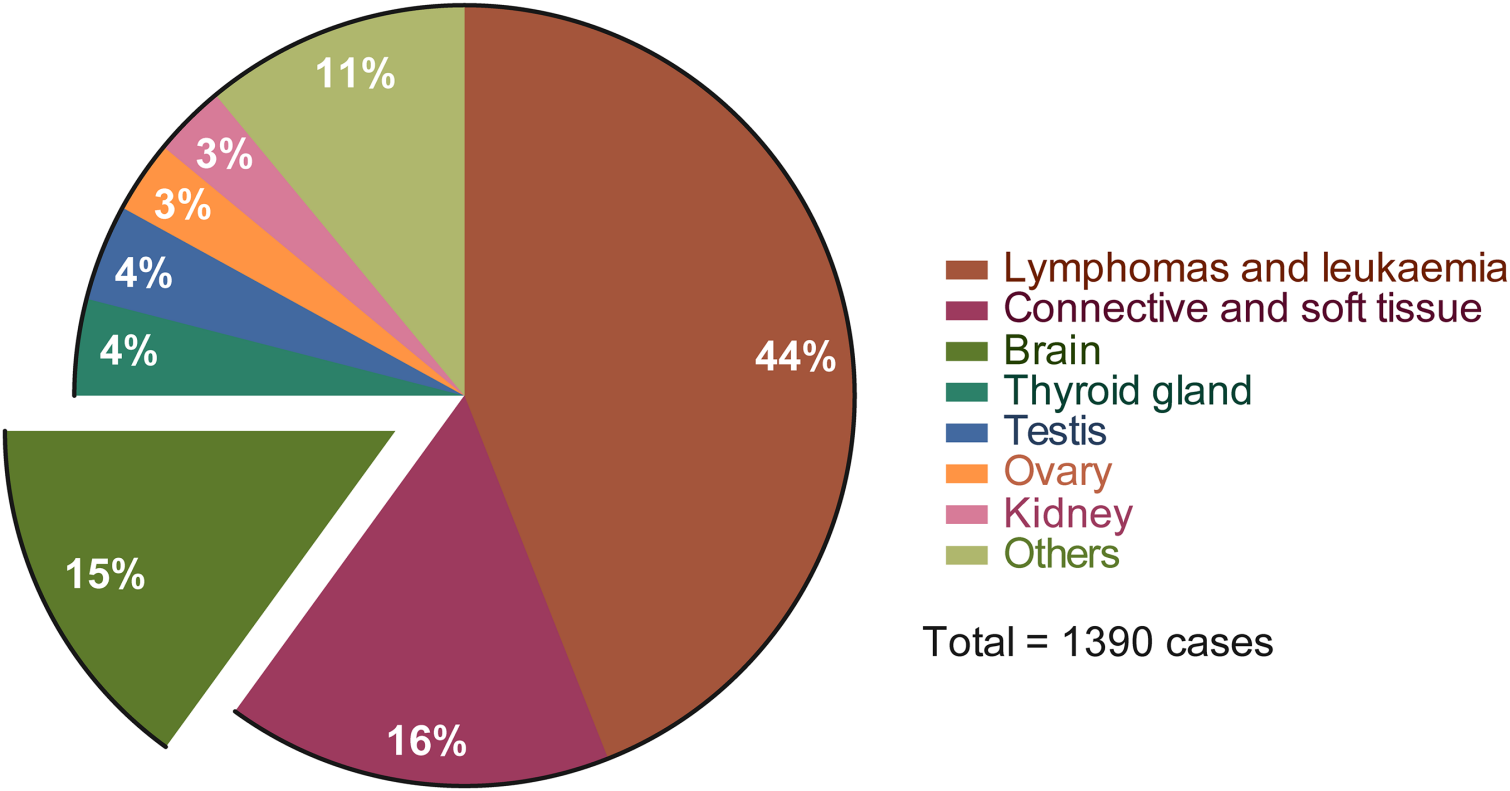
Prevalence of malignancies among children in England. Reported newly diagnosed cases of cancer in England in 2017 among individuals from 4 to 19 years of age [data from the Office for National Statistics].

**Figure 2.**
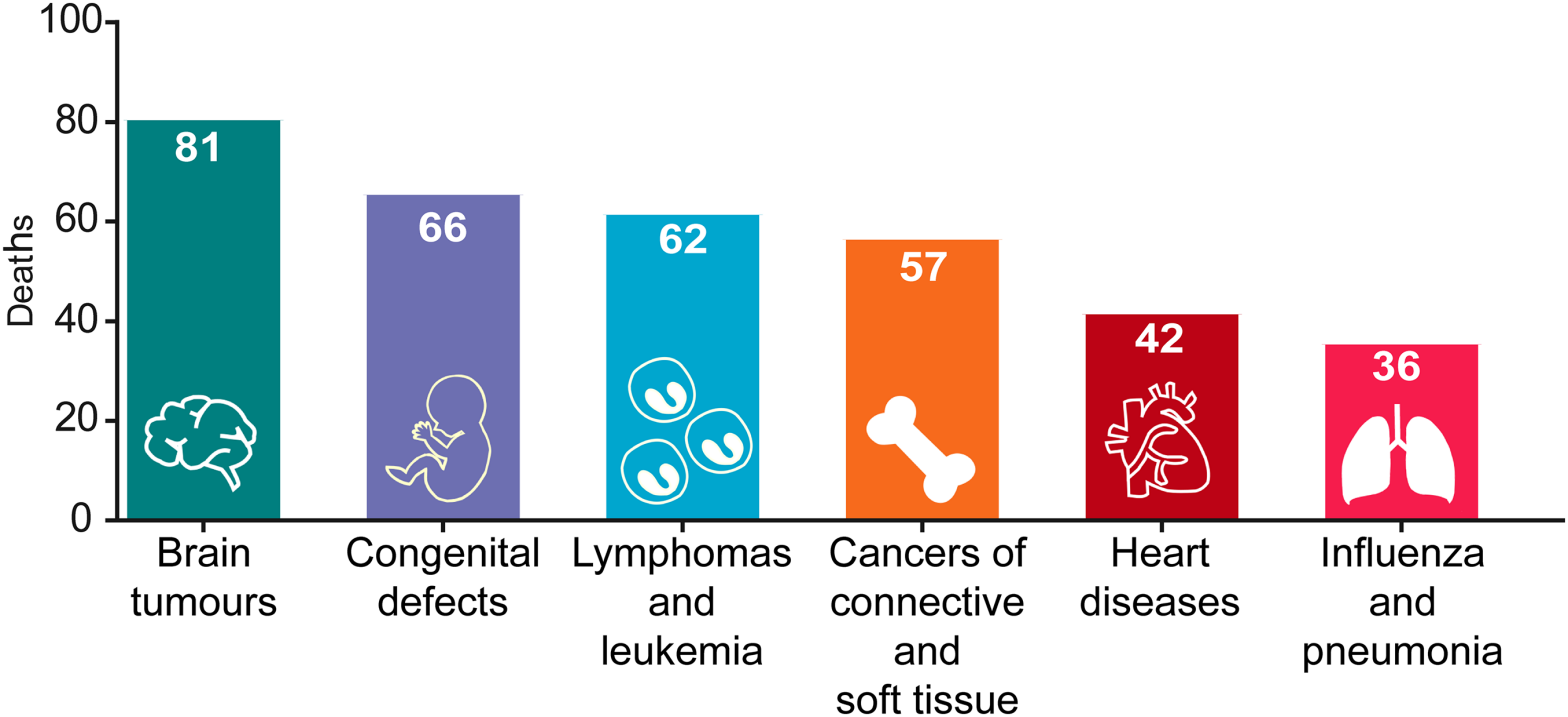
Leading causes of child mortality in England and Wales. Mortality by disease in 2017 in England and Wales among individuals from 4 to 19 years of age [data from the Office for National Statistics].

**Figure 3.**
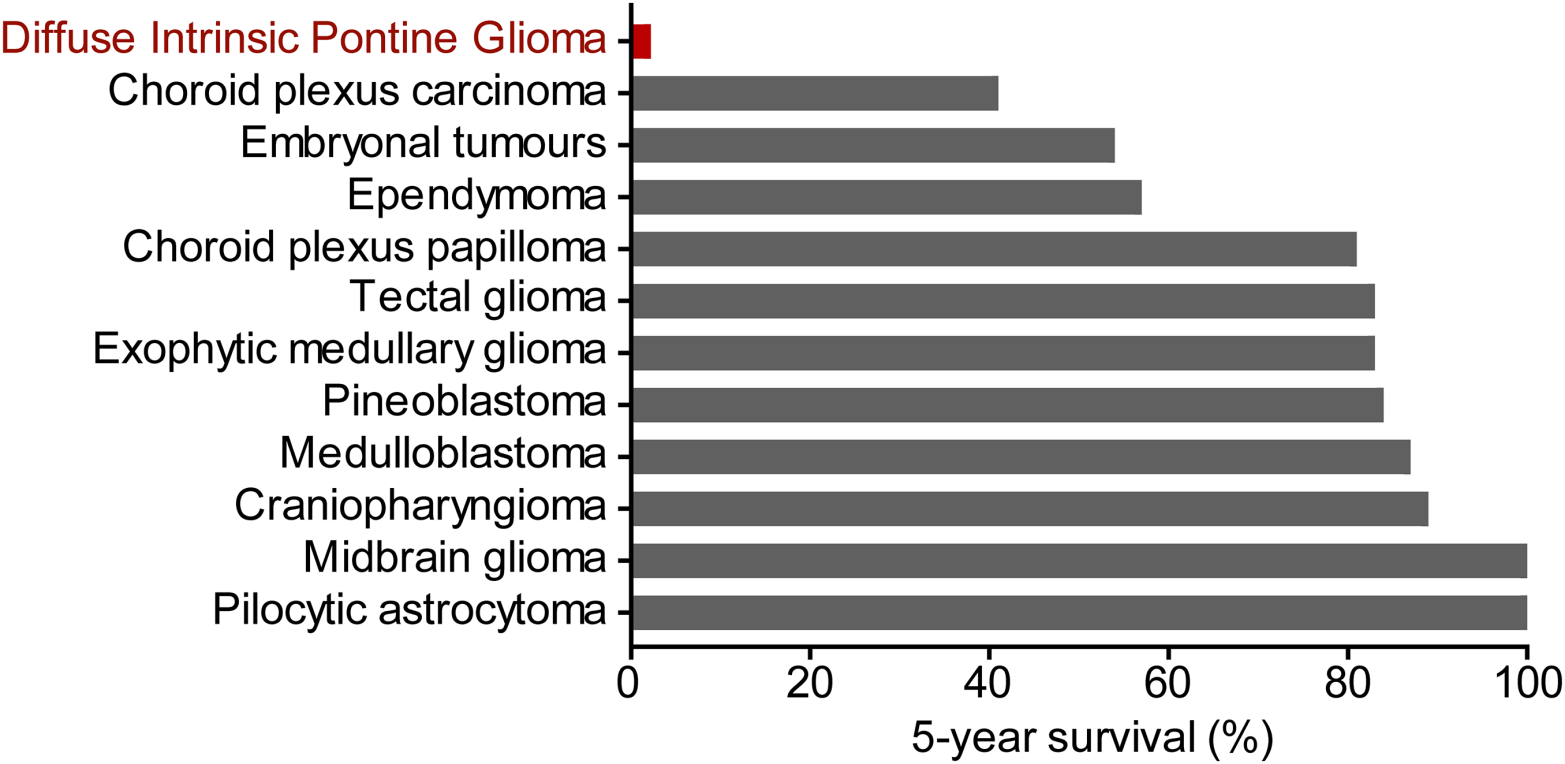
Prognosis of childhood brain tumours. 5-year survival of patients with diffuse intrinsic pontine glioma [5], choroid plexus carcinoma [63], embryonal tumours [64], ependymoma [65], choroid plexus papilloma [63], tectal glioma [66], exophytic medullary glioma [66], pineoblastoma [67], medulloblastoma [68], craniapharyngioma [69], midbrain glioma [66] and pilocytic astrocytoma [70].

Brain tumours comprise 15% of malignancies reported among children (Figure 1) and are responsible for more child mortality than any other disease (Figure 2). In some indications, such as medulloblastoma, a combination of surgical resection and radiotherapy has increased 5-year survival rates to 80% or higher (Figure 3). However, following these treatments survivors often suffer from long term cognitive and sensory decline [1,2]. The prognosis of paediatric brain tumours has lagged behind other cancers in part due to their inaccessibility to treatment, with the blood-brain-barrier being one of the major hurdles for the effectiveness of targeted therapies [3]. In addition, funding for brain tumour research in the U.K., as well as the United States, has been limited historically to just 1% of the national spend on cancer research [3,4].

Diffuse intrinsic pontine glioma (DIPG) is a highly aggressive malignant tumour of the brainstem typically affecting children in the age range of 5–7 years. DIPG accounts for 10% of paediatric brain tumours and is almost uniformly fatal with a median survival of just 11 months (Figure 3) [5]. The affected pons region of the brain controls vital body functions including respiration and sleep that prohibit conventional treatment by surgical resection. Radiotherapy is the current standard of care, but offers only limited short term benefit for DIPG patient survival [6]. Many chemotherapeutic agents, including temozolomide and vincristine, have been tested alongside radiotherapy, but have similarly failed to demonstrate any significant improvement in outcome [7,8]. More research and investment is therefore needed to address DIPG pathophysiology and treatment.

## Genetic basis for DIPG

Historically, the absence of effective treatments and risks associated with biopsy limited the availability of patient material for DIPG research and diagnosis. Consequently, childhood and adult gliomas were long considered similar tumours for classification and treatment [9]. However, the introduction of safe procedures for stereotactic biopsy of DIPG tumours established a new opportunity for molecular characterisation [10]. Overall, paediatric cancers are associated with a reduced mutational burden due to their earlier age of onset [11]. Whole-genome sequencing has further revealed that DIPG is associated with a unique set of genetic mutations. While adult glioma is driven by oncogenic mutations in *IDH1/2*, serine/threonine-protein kinase B-raf (*BRAF*) or fibroblast growth factor receptor (*FGFR*), paediatric cases of DIPG are characterised by mutations in histone proteins as well as developmental signalling proteins [12,13].

The central role of epigenetic reprogramming in DIPG development is highlighted by the presence of heterozygous *H3F3A* or *HIST1H3B* K27M mutations in 80% of all DIPG cases [14,15]. Although quantification of cellular histone proteins indicates that this oncohistone represents only 15% of the histone pool, a broad global change in genome methylation is observed as a result of inhibition of the PRC2 complex by the K27M moiety [16–18]. These epigenetic alterations likely lead to the maintenance of a specific developmental state or pattern of gene expression in DIPG cells that promotes tumour growth [19]. This, along with the narrow window of the age of onset, strongly suggests the dependence of DIPG on a particular developmental process [20,21]. H3K27M mutations are closely paired spatially and temporally to additional clonal driver mutations in one of three categories (i) those affecting *TP53* or cell cycle regulation; (ii) activation of growth factor receptors such as the tyrosine kinase platelet-derived growth factor receptor alpha (*PDGFRA*); (iii) or missense mutations in *ACVR1*, which encodes the receptor serine/threonine kinase ALK2 [22–25]. These are further accompanied by subclonal alterations in accessory drivers such as the PI3K pathway.

Perhaps the most compelling discovery was the identification of *ACVR1* mutations in 25% of DIPG cases. These somatic mutations are largely absent from any other human cancer, with the exception of 1.7% of endometrial cancers [26]. However, identical *ACVR1* mutations are found in the germline in individuals with the monogenic developmental disorder fibrodysplasia ossificans progressiva (FOP), characterised by skeletal abnormalities and disabling heterotopic ossification [27–29]. *ACVR1* mutations in DIPG are in general mutually exclusive with mutations altering *TP53* and *PDGFRA* and associate more strongly with K27M mutations in *HIST1H3B* rather than *H3F3A* [12,13]. Of note, mutant *ACVR1* occurs ubiquitously throughout these DIPG tumours suggesting that mutant *ACVR1 is* an early and obligate partner of H3K27M mutations during clonal evolution [30]. For yet unknown reasons, the occurrence of *ACVR1* mutations in DIPG is also biased towards female over male children [12,13].

**Figure 4.**
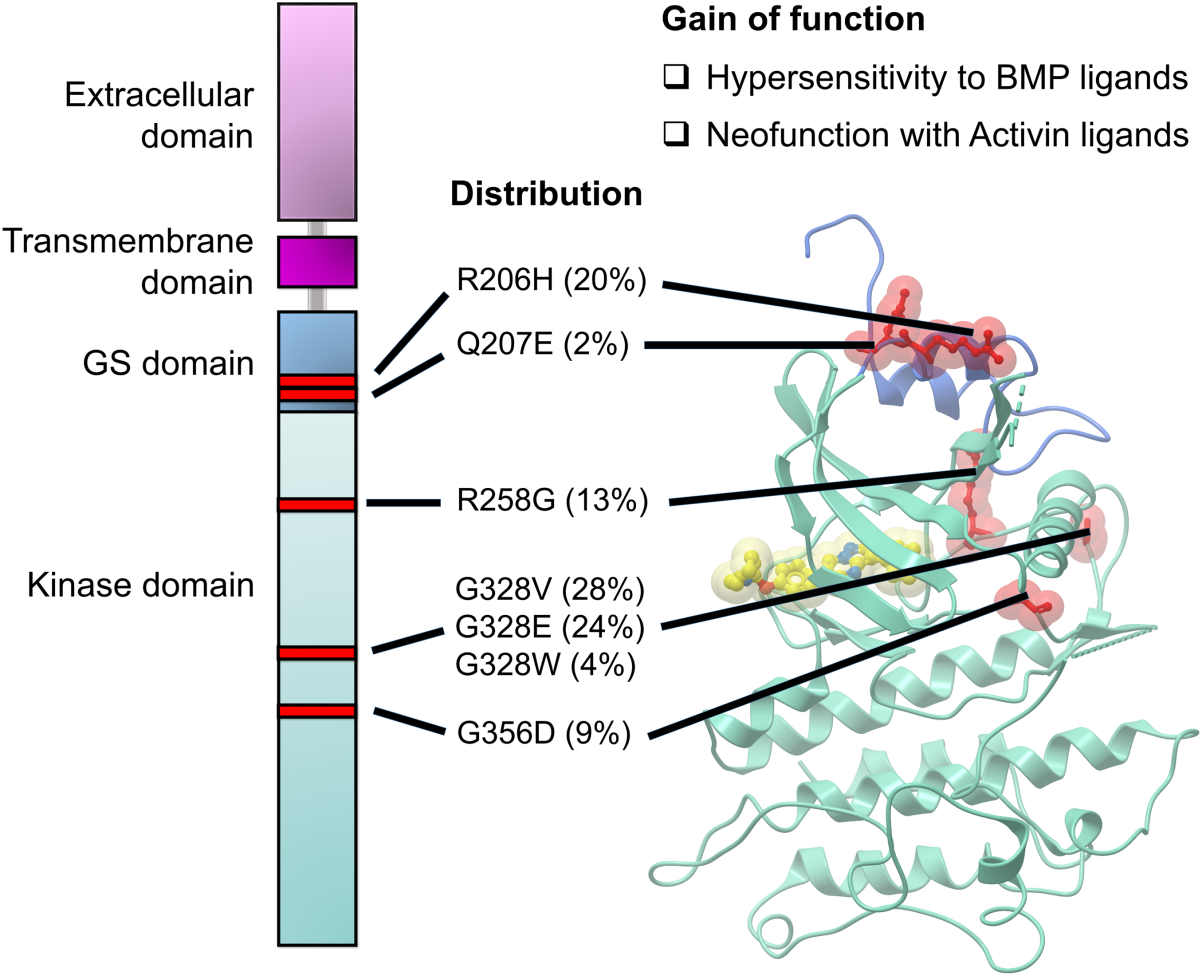
Distribution of ALK2 mutations identified in DIPG. Domain organisation of ALK2 showing the distribution of amino acid positions (red) that are found mutated in DIPG. Sites of mutations (wild-type residues shown as red sticks) are mapped onto the crystal structure (PDB 3H9R [54]) of the wild-type ALK2 GS (dark blue) and kinase (light blue) domains with a summary of their gain of function. The location of the ATP-binding pocket is indicated by the presence of a bound inhibitor molecule (dorsomorphin, shown as yellow sticks). The relative prevalence among the different ALK2 mutations is shown as reported by Taylor et al. [27].

*ACVR1* encodes for the transmembrane type I bone morphogenetic protein (BMP) receptor ALK2, which displays an extracellular ligand-binding domain and an intracellular serine/threonine kinase domain [31]. Secreted BMP ligands bind to ALK2 in a complex with type II BMP receptors, which then activate ALK2 by transphosphorylation of its juxtamembrane glycine-serine rich (GS) region. This, in turn, promotes the recruitment and phosphorylation of transcription factors SMAD1/5/8 by ALK2 leading to induction of BMP-response genes such as *ID1–3* [32–34]. Heterozygous missense mutations occur exclusively in the intracellular GS and kinase domains of the ALK2 protein causing gain of function, including a neofunction through an induced responsiveness to activin ligands, as well as hypersensitivity to canonical BMP ligands (Figure 4) [35,36]. BMP signalling during neurodevelopment is a recognised driver of astrocytic differentiation, consistent with the histology of ALK2 mutant DIPG tumours [27,37]. Expression of ID1–3 is also a known mediator of gliomagenesis [38–41].

## ALK2 represents a novel druggable target for DIPG

The most commonly mutated targets in DIPG, histone H3 and p53, are not directly druggable, although beneficial effects have been observed in DIPG models using inhibitors of epigenetic regulatory proteins including EZH2 [42], KDM6A/B [43] and BRD2/4 [44]. However, classical drug targets are represented by the protein kinases PDGFRA and ALK2. Alterations in these genes, including amplifications and activating mutations, typically occur in a mutually exclusive manner, but together are found in more than half of all DIPG cases. Clinical trials using approved drugs targeting PDGFRA are already in progress [45].

DIPG research further benefits from a pre-existing library of ALK2 inhibitors developed over the past decade due to the prior linkage of mutant ALK2 to FOP [27]. A pyrazolo[1,5-a]pyrimidine inhibitor series was developed following the discovery of dorsomorphin as a small molecule capable of inducing dorsalisation of zebrafish [46]. Potent derivatives, including LDN-193189 and LDN-212854, were subsequently developed showing striking efficacy in FOP mouse models [47,48]. The second series of 3,5-diaryl-pyridines, including K02288 and LDN-214117, was identified through biochemical screens using recombinant ALK2 protein [49,50]. The diversity of ALK2 inhibitors was further increased with the recent discovery of novel quinazolinones [51], as well as bis-heteroaryl pyrazoles [52]. More significantly, several ALK2 inhibitors are entering into clinical development for FOP, including BLU-782 and BCX9250 [53]. The binding affinity of the inhibitors appears independent of the specific mutation in ALK2, suggesting that any drug molecule would have general application against mutant ALK2 [50]. This likely reflects the fact that the mutations occur outside the ATP-binding pocket at sites that regulate the kinase activation state (Figure 4) [54].

FOP patients do not develop DIPG showing that mutant ALK2 is not sufficient to drive tumour formation alone. However, a viral infection of neurospheres has demonstrated significant cooperation between mutant ALK2 and the oncohistone H3.1K27M as well as a strong contribution of ALK2 towards gliomagenesis, with observed effects on tumour initiation, proliferation and survival [55]. Importantly, the ALK2 inhibitors LDN-193189, LDN-212854 and LDN-214117 have all demonstrated suitable brain penetration, tolerability and pharmacokinetic properties for use in orthotopic *in vivo* models of DIPG [55,56]. Indeed, beneficial effects were observed for all three inhibitors in mice, including a 15-day increase in survival either using patient-derived xenografts [56] or viral infection models of DIPG [55]. Together, these data support the further development of ALK2 inhibitors for clinical investigation in DIPG. Additional work to evaluate potential combination drug treatments would be advantageous as the efficacy of ALK2 inhibitors varies across different patient-derived cell lines, potentially reflecting their different genetic backgrounds [56]. Detailed toxicology studies would also be needed to evaluate safe dosing of ALK2 inhibitors in children.

## Rethinking business models to enable drug development for DIPG

Paediatric brain tumours such as DIPG present a hugely challenging indication for traditional pharma business models due to the population size, high failure rate, tumour location and age of onset. Only 40 cases of DIPG are typically diagnosed each year in the U.K. and only 10 of these may be expected to express mutant ALK2 [57]. Thus, the potential for any financial return for investors is limited. Consequently, clinical trials for paediatric brain tumours generally involve the repurposing of existing medicines rather than new investigational drugs. To date more than 250 clinical trials exploring chemotherapeutic agents in DIPG have failed, reflecting the unique genomic landscape of these tumours compared with other human cancers [58]. Failure is further exacerbated by the fact that most drugs developed by the pharmaceutical industry are designed not to cross the blood-brain barrier to strengthen their safety profile; this includes ongoing programmes on the most relevant targets, including epigenetic modulators as well as ALK2 inhibitors in clinical development for FOP. Finally, a risk adverse industry is traditionally biased towards developing new treatments for adult indications rather than targeting new investigational drugs towards sick children.

An innovative approach is needed to overcome these obstacles and to reduce risk. One example is provided by M4K Pharma (‘Meds4Kids’), which was founded specifically in the first instance to develop the existing preclinical ALK2 inhibitors into a brain penetrant drug candidate for DIPG [59]. M4K Pharma operates as a virtual biotech that aims to attract, align and aggregate diffuse academic and industry research into a robust drug development programme. To achieve this goal it is testing an open science model that encourages collaboration without restrictions. The company itself is owned by the Agora Open Science Trust for which the beneficiaries are ‘open science and the public good’ [59]. Instead of filing for patents the company encourages open disclosure of its data that establishes it as ‘prior art’. Such disclosure prevents third parties from filing later patents in the same chemical space that would otherwise block the company from marketing its own drug. However, unlike patents, prior art cannot be used to exclude others from working in the same chemical space. Thus, disclosure of prior art presents a mechanism to balance the rewards of unrestricted collaboration with the mitigation of risk from the competition. If successful, M4K Pharma would benefit from government incentives for orphan paediatric indications such as the regulatory exclusivity period for marketing. This can extend for up to 12 years within the European Union and therefore compares favourably with the term remaining on most patents upon marketing [59].

Of note, the open collaborative model of M4K Pharma has proven attractive to governments, disease foundations and even industry partners. For example, funding for the ALK2 programme has come from grants from the Ontario Institute for Cancer Research and The Brain Tumour Charity. In addition, further in-kind support has been provided by Charles River Laboratories, Reaction Biology and GL Chemtec [59]. Industry involvement fulfils a desire for corporate social responsibility while establishing an opportunity for staff engagement and exposure. The collaborative M4K Pharma model may also help to leverage future institutional support as well as charitable or government funding towards the costs of early-stage proof of concept clinical trials.

By securing grant funding rather than venture capital, M4K Pharma aims to cap any future drug pricing to affordable levels, while returning any profit to charitable causes, including new research programmes. This contrasts with the model adopted by the Cystic Fibrosis Foundation (CFF), which invested $75 million in an exclusive partnership with Vertex Pharmaceuticals to develop the precision medicine ivacaftor and the subsequent combination drug Orkambi. While CFF was successful in selling its right to future royalties for $3.3 billion, it did not retain rights to constrain pricing or rights to guarantee access for patients. Ivacaftor eventually launched in the US at a cost of $311 000 per patient per year [60], while Orkambi currently remains unavailable in England through the National Health Service due to a long-standing dispute over pricing [61]. In contrast, the M4K Pharma model aims to negotiate with commercial partners later in development when a more validated asset may provide a stronger position to commit potential manufacturing and distribution partners to affordable pricing. This position may be further strengthened in the United States where drug approvals for rare paediatric diseases also earn companies a priority review voucher that can be potentially traded for millions of dollars [62].

Overall, the open science model itself presents an experiment in drug development. At the very least, an open collaborative approach should help to reduce duplication and offer rapid learning from interim failures as well as successes.

## Conclusions

The current standard of care in conditions such as DIPG is failing to impact upon patient survival compelling the scientific community to consider new approaches to derive effective medicines. Whole-genome sequencing has newly described the molecular basis for many paediatric brain tumours revealing striking differences to adult cancers. These studies have uncovered new links to developmental signalling pathways, as well as important roles for chromatin modifiers. A major issue remains the apparent heterogeneity of both adult and childhood gliomas [3]. Thus, tumour types are divided into ever smaller subcategories and patient populations presenting yet greater challenges for traditional economic models of drug development. The highly aggressive nature of these tumours may demand the use of well-tolerated combination treatments. Thus, researchers are tasked with the development of a toolkit of compounds and/or treatment modalities to address the specific genetic liabilities of different brain tumours. The rather unique association of *ACVR1* mutations with DIPG tumours highlights this growth factor receptor as a promising target for future clinical investigation.

**Perspectives**

- The prognosis for patients diagnosed with brain tumours remains abysmal and largely unchanged over the past 30 years. The paediatric brain tumour DIPG, in particular, is almost uniformly fatal with a median survival time of just 11 months following diagnosis.
- Current treatments rely on radiotherapy, chemotherapy and surgical resection. However, these approaches can cause lifelong and debilitating side effects. Moreover, surgical resection is not possible for brainstem tumours such as DIPG. A new understanding of the epigenetic and genetic causes of paediatric brain tumours has recently emerged revealing potential novel targets for drug development, including the growth factor receptor ALK2, encoded by the gene ACVR1.
- The high risks and low financial rewards associated with developing new treatments for paediatric brain tumours present a huge challenge for the future pharmaceutical industry. Nonetheless, governments have recognised the need for more investment in this research area and significant efforts are ongoing across the world in both academia and industry. More open and collaborative ventures, such as that currently being trialled by M4K Pharma, may help to align these diffuse efforts into collective programmes that can deliver new orphan medicines and relieve the suffering of cancer patients.

## Abbreviations

*ACVR1*: activin A receptor type 1
ALK2: activin receptor-like kinase-2
BMP: bone morphogenetic protein
*BRAF*: serine/threonine-protein kinase B-raf
BRD2/4: bromodomain-containing proteins 2 and 4
CFF: Cystic Fibrosis Foundation
DIPG: diffuse intrinsic pontine glioma
EZH2: histone-lysine *N*-methyltransferase enhancer of zeste homologue 2
*FGFR*: fibroblast growth factor receptor
FOP: fibrodysplasia ossificans progressiva
GS: glycine-serine rich
*H3F3A*: histone H3.3
*HIST1H3B*: histone H3.1
*ID1–3*: Inhibitor of differentiation 1–3
*IDH1/2*: isocitrate dehydrogenase 1 and 2
KDM6A/B: lysine-specific demethylases 6A and 6B
M4K: Meds4Kids
*PDGFRA*: platelet-derived growth factor receptor alpha
PRC2: polycomb repressive complex 2
SMAD1/5/8: mothers against decapentaplegic homologues 1, 5 and 8
*TP53*: cellular tumour antigen p53
